# On the identification of twinning in body-centred cubic nanoparticles†
†Electronic supplementary information (ESI) available: Additional simulation results (PDF). See DOI: 10.1039/d0nr06957d


**DOI:** 10.1039/d0nr06957d

**Published:** 2020-10-23

**Authors:** Elizabeth R. Hopper, Christina Boukouvala, Duncan N. Johnstone, John S. Biggins, Emilie Ringe

**Affiliations:** a Department of Materials Science and Metallurgy , University of Cambridge , 27 Charles Babbage Road , Cambridge , CB3 0FS , UK . Email: er407@cam.ac.uk; b Department of Earth Sciences , University of Cambridge , Downing Street , Cambridge , CB2 3EQ , UK; c Department of Chemical Engineering and Biotechnology , University of Cambridge , Philippa Fawcett Drive , Cambridge , CB3 0AS , UK; d Department of Engineering , University of Cambridge , Trumpington Street , Cambridge , CB2 1PZS , UK

## Abstract

Many metals and alloys, including Fe and W, adopt body-centred cubic (BCC) crystal structures and nanoparticles of these metals are gaining significant scientific and industrial relevance. Twinning has a marked effect on catalytic activity, yet there is little evidence for or against the presence of twinning in BCC nanoparticles. Here, we explore the potential shapes of twinned BCC nanoparticles, and predict their electron microscopy and diffraction signatures. BCC single crystal and twinned shapes often appear similar and diffraction patterns along common, low-index zone axes are often indistinguishable, casting doubt on many claims of single crystallinity. We conclude by outlining how nanoparticles can be characterized to conclusively prove the presence or absence of twinning.

## Abstract 2

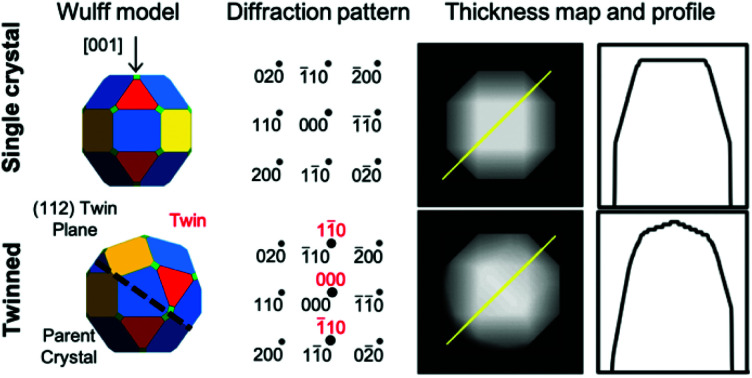
This Communication describes diagnostic approaches to identify BCC twinning based on shape as well as electron microscopy and diffraction signatures.

## 

About one third of metallic elements crystallise in a body-centred cubic (BCC) structure, including the transition metals Cr, Mo, W and Fe. Nanoparticles (NPs) of these metals are finding applications in strain sensing,^1^ catalysis,^2^–^5^ and, for Fe, medical diagnosis, treatment, and electronic media.^5^ Twinning and grain boundaries are known to affect catalytic activity,^6^–^10^ yet there is little recorded evidence for or against the presence of twinning in BCC NPs.

Catalytic properties are controlled by NP shape, composition, and crystallinity. Twinning in NPs, *i.e.* the presence of one or more planar crystallographic defects, leads to not only novel shapes but also strain that influences catalytic properties.^6^–^8^ For instance, twinned icosahedral Pd and Pt–Ni NPs have higher activities than single crystal octahedra for formic acid oxidation and oxygen reduction, respectively, despite both being bound exclusively by {111} facets;^7^,^8^ this is attributed to strain bestowed by the twin boundaries. Furthermore, the presence and density of related, strain-inducing defects such as grain boundaries increase the catalytic activity of Au for the reduction of CO_2_ to CO and of Cu for the electrochemical reduction of CO to ethanol, acetate, propanol, and ethylene.^9^,^10^ Scanning electrochemical cell microscopy directly confirmed the increase in local activity at grain boundaries of Au electrodes for the reduction of CO_2_.^11^ The characterization, understanding, and control of twinning and polycrystallinity is, therefore, crucial for designing catalysts.

Fuelled by their exciting catalytic and magnetic properties, an increasing number of syntheses of (presumed) single-crystalline NPs of Fe^12^–^14^ and W,^15^,^16^ both BCC, are emerging. The NPs have diameters as small as 10 nm, and their single-crystallinity is typically supported by shape observation and a single low-index electron diffraction pattern, which we demonstrate is insufficient evidence.

Why has seemingly no one looked for twinning in BCC NPs, and why are such unconvincing arguments on single crystallinity accepted? Likely, it is because BCC metals do not readily form growth twins in the bulk, and, therefore, it is assumed they are not present at the nanoscale. The argument here is that BCC is not like face-centred cubic (FCC) systems, where {111} growth twins are ubiquitous in bulk and nanostructures.^17^ Until recently, BCC might have instead been said to behave like hexagonal close-packed (HCP) structures, both having rare bulk growth twins. However, very recently, following the steps of Ohno and Yamauchi,^18^ we showed that growth twins behave differently at the nanoscale, demonstrating that nearly half (48%) of solution-grown NPs of HCP Mg are twinned.^19^ Further, BCC structures readily produce deformation twins on the {112} planes, seen upon shearing of nanopillars^20^ and in bulk specimens.^21^ In this light, we question the absence of twinning in BCC NPs.

The shape of a freestanding (*e.g.*, solution-grown) crystal is related to the relative surface energy of its facets following the Wulff construction; in real systems growth velocities are used instead of thermodynamic energies to include kinetic effects.^22^ The mathematical proportionality between the surface energy/growth velocity and the distance from a facet to the NP centre implies that facets of low energy/growth velocity dominate the structure. Wulff constructions for twinned crystals are built from a parent crystal and one (or more) twins, all truncated and joined by one (or more) shared twin plane.^22^,^23^ In BCC, Fig. 1a, we assume twinning on the {112} planes and low growth velocities for the {100}, {110}, {111} and {112} facets with their relative values depending on reaction conditions.^12^,^24^,^25^

**Fig. 1:**
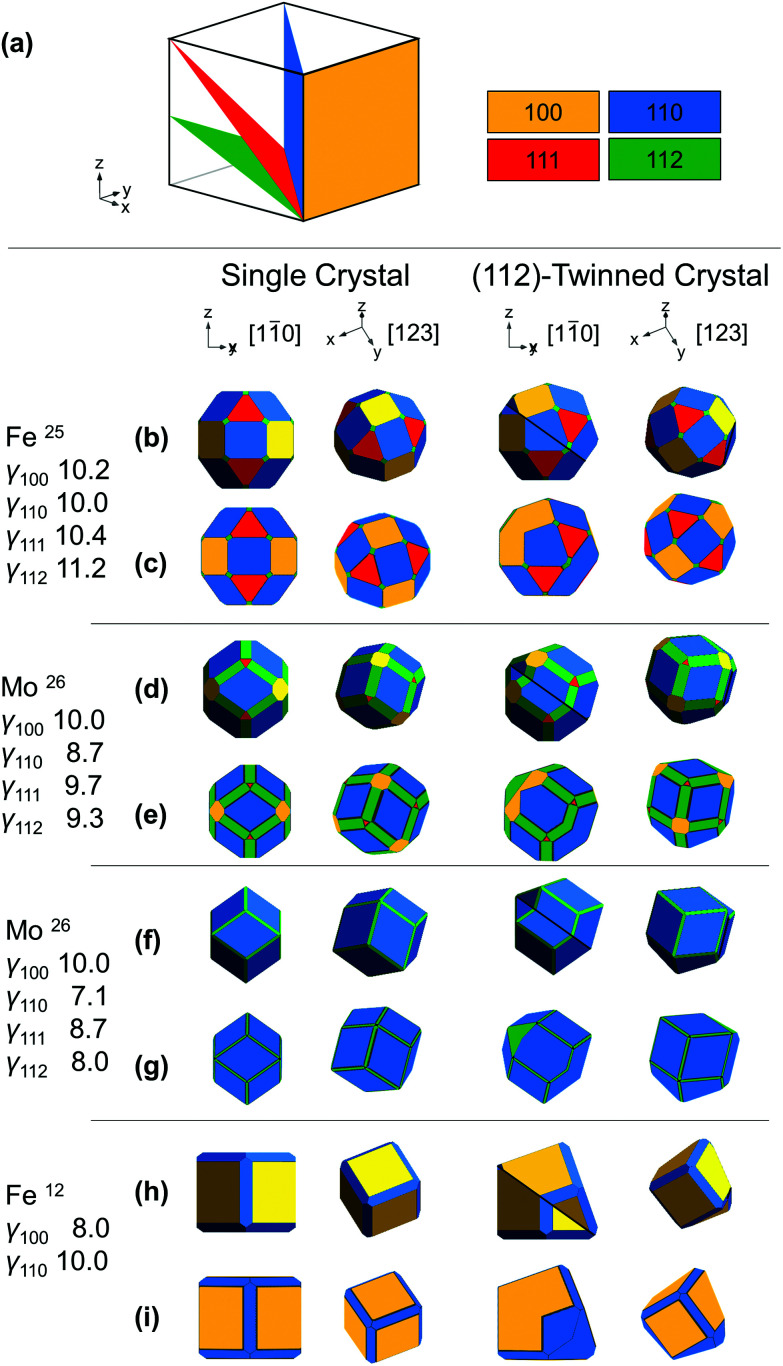
Shapes of BCC NPs. (a) Dense crystallographic planes in BCC and their color-coding. (b, d, f and h) Analytical thermodynamic and (c, e, g and i) numerical kinetic shapes from the surface energies/growth velocities listed; twin planes are shown as a black line, viewing directions are [11[combining macron]0] or [123]; these and the *x*, *y*, *z* directions refer to the parent crystal, on, for [11[combining macron]0], the bottom left of and, for [123], behind the crystal.

To establish realistic shapes and surface energies, we modelled shapes inspired by numerical and experimental results. Wulffmaker^26^ and Crystal Creator^19^,^27^ were used to predict single crystalline NP shapes based on the relative surface energies or growth velocities reported for Fe and Mo^24^,^25^ (Fig. 1b–g). Complementarily, relative surface energies were extracted from matching published NP shapes from ref^12^ (Fig. 1h and i). The relative surface energies/growth velocities differ widely under varying experimental conditions, and in turn so do the shapes obtained. In Fe NPs, truncated cuboctahedra have been observed and modelled;^24^ truncated nanocubes have also been seen.^12^ In Mo, first-principles calculations and bond-cutting calculations yield differently proportioned Bijinski dodecahedra.^25^

Wulffmaker and Crystal Creator were then modified to model (112)-twinned BCC crystals, using surface energies/growth velocities mapped on those of single crystals. The twinned shapes produced are remarkably similar to single crystal shapes (Fig. 1).

This similarity between single crystal and twinned shapes undermines shape-based experimental identification of twinned BCC NPs. Simple shape characterization approaches such as scanning electron microscopy (SEM) or atomic force microscopy (AFM) reveal out-of-plane topography; however, given the typically small size (<50 nm) of catalytically relevant NPs and experimental edge/corner rounding, low resolution and shape similarities limit their use. High-angle annular dark field scanning transmission electron microscopy (HAADF-STEM) offers improved resolution over SEM and more straightforwardly interpretable images than TEM; it produces thickness projections such as those predicted in Fig. 2 and S1.† The cuboctahedral single crystal and related twinned NPs look virtually identical along the [001], [110] and [111] zone axes, for instance (Fig. 2). Taking a similar shape approach, one could use STEM-HAADF 3D tomography, yet again rounding and small sizes will cause issues unless atomic resolution is achieved.

**Fig. 2:**
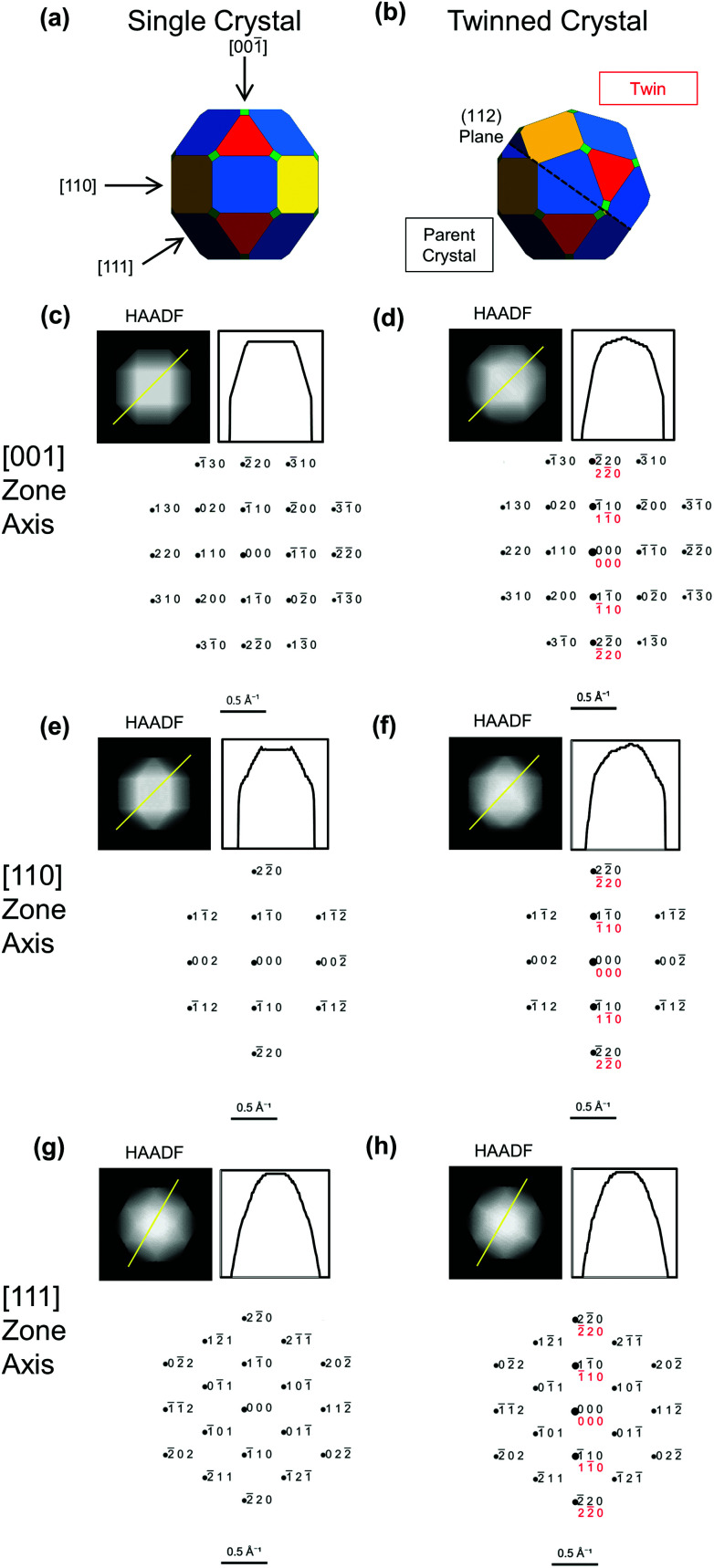
Simulated images and diffraction patterns for BCC single crystal (left) and twinned NPs (right) from Fig. 1b and c, reproduced in (a and b). (c–h) Projected thickness maps as a proxy for HAADF-STEM images, thickness profile along the yellow line, and simulated diffraction patterns, with parent in black and twin in red. Diffraction spots areas scale with intensity.

The analytical model (Wulffmaker-based) is purely thermodynamic, whereas kinetically enhanced growth at re-entrant corners was included in the numerical Crystal Creator approach. This effect likely gives more accurate predictions of experimental shapes, as demonstrated in twinned FCC NPs, where experimentally observed bipyramids form kinetic products without thermodynamically predicted re-entrant notches.^22^ While kinetic effects can vary depending on growth conditions, here their extent makes little difference in the perceived crystal shape (Fig. 1 and S2†), and twinning only marginally disturbs the facet surface area ratio (Table S1†).

The shape differences between twinned and single-crystalline NPs are often too subtle for definitive identification, so characterization should instead rely on probing the symmetry of atomic packing. This approach can clearly indicate the presence of the twin defect, either *via* diffraction or direct lattice imaging. Diffraction patterns are indeed common evidence of the crystallinity of novel shapes.^17^,^28^ Due to the different orientations of the parent and twinned crystals, the superposition of their (distinct) diffraction patterns can differ from that of single crystals. For instance, distinguishable patterns are obtained along [101[combining macron]] for (111)-twinned FCC crystals (Fig. S3†) and [101] for (112)-twinned BCC crystals (Fig. 3).

**Fig. 3:**
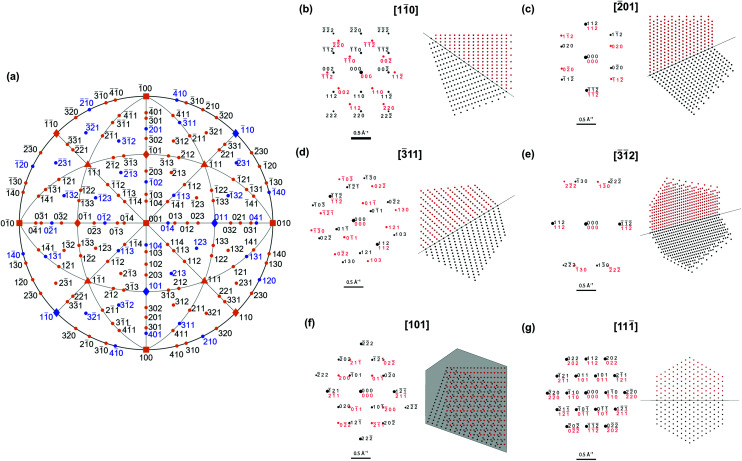
Diffraction patterns diagnostic of a BCC NP twinned on the (112) plane. (a) Stereographic projection with directions showing diagnostic and indistinguishable symmetries coloured blue and orange, respectively. Examples of (b–f) diagnostic and (g) indistinguishable diffraction patterns and atomic arrangements. Black and red colouring indicate the parent and twinned crystal, respectively.

However, diagnostic diffraction patterns are rare along low-index directions in BCC twins. The most straightforward direction along which to record a diffraction pattern is with the electron beam perpendicular to the support film and/or one of the facets. Fan *et al.*^12^ and Wang *et al.*^29^ use the square symmetry of the [001] pattern from a NP to argue for single crystallinity in Fe and W, respectively. Simulated diffraction patterns (Fig. 2c and d) show identical ) show identical 〈001〉 symmetry between twinned and single crystal; the only distinguishing feature is a more intense line along [11̄0], that is in fact hinted in Fan 001) show identical 〈001〉 symmetry between twinned and single crystal; the only distinguishing feature is a more intense line along [11̄0], that is in fact hinted in Fan symmetry between twinned and single crystal; the only distinguishing feature is a more intense line along [11[combining macron]0], that is in fact hinted in Fan *et al.*'s (incorrectly indexed) pattern. Intensities, however, carry large uncertainties: diffraction spots are weak for small NPs and perfect zone axis alignment is difficult to achieve.

A systematic survey of diffraction patterns (Fig. 3, Table S2†) reveals that the symmetry of most patterns from low-index directions (defined as *h* + *k* + *l* ≤ 6 and *h*, *k*, *l* ≤ 4) would indeed not reveal twinning in a BCC NP, potentially leading to incorrect classification. None of the ≤ 4) would indeed not reveal twinning in a BCC NP, potentially leading to incorrect classification. None of the 〈100〉 and 〈111〉 orientations and only 6 of the 12 〈110〉 orientations lead to diagnostic pattern symmetry. Thus, of the 26 probable orientations of a single crystal truncated cuboctahedron on a substrate, only 6 give diffraction patterns with symmetries different from that of a twinned NP. For higher order index directions, requiring careful tilting, 12 of the 36 〈210〉, 12 of the 24 〈311〉, 12 of the 36 〈410〉 and 24 of the 48 〈321〉directions produce distinguishable patterns. This may be why twinned BCC NPs have never been reported.100 ≤ 4) would indeed not reveal twinning in a BCC NP, potentially leading to incorrect classification. None of the 〈100〉 and 〈111〉 orientations and only 6 of the 12 〈110〉 orientations lead to diagnostic pattern symmetry. Thus, of the 26 probable orientations of a single crystal truncated cuboctahedron on a substrate, only 6 give diffraction patterns with symmetries different from that of a twinned NP. For higher order index directions, requiring careful tilting, 12 of the 36 〈210〉, 12 of the 24 〈311〉, 12 of the 36 〈410〉 and 24 of the 48 〈321〉directions produce distinguishable patterns. This may be why twinned BCC NPs have never been reported. and ≤ 4) would indeed not reveal twinning in a BCC NP, potentially leading to incorrect classification. None of the 〈100〉 and 〈111〉 orientations and only 6 of the 12 〈110〉 orientations lead to diagnostic pattern symmetry. Thus, of the 26 probable orientations of a single crystal truncated cuboctahedron on a substrate, only 6 give diffraction patterns with symmetries different from that of a twinned NP. For higher order index directions, requiring careful tilting, 12 of the 36 〈210〉, 12 of the 24 〈311〉, 12 of the 36 〈410〉 and 24 of the 48 〈321〉directions produce distinguishable patterns. This may be why twinned BCC NPs have never been reported.111 ≤ 4) would indeed not reveal twinning in a BCC NP, potentially leading to incorrect classification. None of the 〈100〉 and 〈111〉 orientations and only 6 of the 12 〈110〉 orientations lead to diagnostic pattern symmetry. Thus, of the 26 probable orientations of a single crystal truncated cuboctahedron on a substrate, only 6 give diffraction patterns with symmetries different from that of a twinned NP. For higher order index directions, requiring careful tilting, 12 of the 36 〈210〉, 12 of the 24 〈311〉, 12 of the 36 〈410〉 and 24 of the 48 〈321〉directions produce distinguishable patterns. This may be why twinned BCC NPs have never been reported. orientations and only 6 of the 12  ≤ 4) would indeed not reveal twinning in a BCC NP, potentially leading to incorrect classification. None of the 〈100〉 and 〈111〉 orientations and only 6 of the 12 〈110〉 orientations lead to diagnostic pattern symmetry. Thus, of the 26 probable orientations of a single crystal truncated cuboctahedron on a substrate, only 6 give diffraction patterns with symmetries different from that of a twinned NP. For higher order index directions, requiring careful tilting, 12 of the 36 〈210〉, 12 of the 24 〈311〉, 12 of the 36 〈410〉 and 24 of the 48 〈321〉directions produce distinguishable patterns. This may be why twinned BCC NPs have never been reported.110 ≤ 4) would indeed not reveal twinning in a BCC NP, potentially leading to incorrect classification. None of the 〈100〉 and 〈111〉 orientations and only 6 of the 12 〈110〉 orientations lead to diagnostic pattern symmetry. Thus, of the 26 probable orientations of a single crystal truncated cuboctahedron on a substrate, only 6 give diffraction patterns with symmetries different from that of a twinned NP. For higher order index directions, requiring careful tilting, 12 of the 36 〈210〉, 12 of the 24 〈311〉, 12 of the 36 〈410〉 and 24 of the 48 〈321〉directions produce distinguishable patterns. This may be why twinned BCC NPs have never been reported. orientations lead to diagnostic pattern symmetry. Thus, of the 26 probable orientations of a single crystal truncated cuboctahedron on a substrate, only 6 give diffraction patterns with symmetries different from that of a twinned NP. For higher order index directions, requiring careful tilting, 12 of the 36  ≤ 4) would indeed not reveal twinning in a BCC NP, potentially leading to incorrect classification. None of the 〈100〉 and 〈111〉 orientations and only 6 of the 12 〈110〉 orientations lead to diagnostic pattern symmetry. Thus, of the 26 probable orientations of a single crystal truncated cuboctahedron on a substrate, only 6 give diffraction patterns with symmetries different from that of a twinned NP. For higher order index directions, requiring careful tilting, 12 of the 36 〈210〉, 12 of the 24 〈311〉, 12 of the 36 〈410〉 and 24 of the 48 〈321〉directions produce distinguishable patterns. This may be why twinned BCC NPs have never been reported.210 ≤ 4) would indeed not reveal twinning in a BCC NP, potentially leading to incorrect classification. None of the 〈100〉 and 〈111〉 orientations and only 6 of the 12 〈110〉 orientations lead to diagnostic pattern symmetry. Thus, of the 26 probable orientations of a single crystal truncated cuboctahedron on a substrate, only 6 give diffraction patterns with symmetries different from that of a twinned NP. For higher order index directions, requiring careful tilting, 12 of the 36 〈210〉, 12 of the 24 〈311〉, 12 of the 36 〈410〉 and 24 of the 48 〈321〉directions produce distinguishable patterns. This may be why twinned BCC NPs have never been reported., 12 of the 24  ≤ 4) would indeed not reveal twinning in a BCC NP, potentially leading to incorrect classification. None of the 〈100〉 and 〈111〉 orientations and only 6 of the 12 〈110〉 orientations lead to diagnostic pattern symmetry. Thus, of the 26 probable orientations of a single crystal truncated cuboctahedron on a substrate, only 6 give diffraction patterns with symmetries different from that of a twinned NP. For higher order index directions, requiring careful tilting, 12 of the 36 〈210〉, 12 of the 24 〈311〉, 12 of the 36 〈410〉 and 24 of the 48 〈321〉directions produce distinguishable patterns. This may be why twinned BCC NPs have never been reported.311 ≤ 4) would indeed not reveal twinning in a BCC NP, potentially leading to incorrect classification. None of the 〈100〉 and 〈111〉 orientations and only 6 of the 12 〈110〉 orientations lead to diagnostic pattern symmetry. Thus, of the 26 probable orientations of a single crystal truncated cuboctahedron on a substrate, only 6 give diffraction patterns with symmetries different from that of a twinned NP. For higher order index directions, requiring careful tilting, 12 of the 36 〈210〉, 12 of the 24 〈311〉, 12 of the 36 〈410〉 and 24 of the 48 〈321〉directions produce distinguishable patterns. This may be why twinned BCC NPs have never been reported., 12 of the 36  ≤ 4) would indeed not reveal twinning in a BCC NP, potentially leading to incorrect classification. None of the 〈100〉 and 〈111〉 orientations and only 6 of the 12 〈110〉 orientations lead to diagnostic pattern symmetry. Thus, of the 26 probable orientations of a single crystal truncated cuboctahedron on a substrate, only 6 give diffraction patterns with symmetries different from that of a twinned NP. For higher order index directions, requiring careful tilting, 12 of the 36 〈210〉, 12 of the 24 〈311〉, 12 of the 36 〈410〉 and 24 of the 48 〈321〉directions produce distinguishable patterns. This may be why twinned BCC NPs have never been reported.410 ≤ 4) would indeed not reveal twinning in a BCC NP, potentially leading to incorrect classification. None of the 〈100〉 and 〈111〉 orientations and only 6 of the 12 〈110〉 orientations lead to diagnostic pattern symmetry. Thus, of the 26 probable orientations of a single crystal truncated cuboctahedron on a substrate, only 6 give diffraction patterns with symmetries different from that of a twinned NP. For higher order index directions, requiring careful tilting, 12 of the 36 〈210〉, 12 of the 24 〈311〉, 12 of the 36 〈410〉 and 24 of the 48 〈321〉directions produce distinguishable patterns. This may be why twinned BCC NPs have never been reported. and 24 of the 48  ≤ 4) would indeed not reveal twinning in a BCC NP, potentially leading to incorrect classification. None of the 〈100〉 and 〈111〉 orientations and only 6 of the 12 〈110〉 orientations lead to diagnostic pattern symmetry. Thus, of the 26 probable orientations of a single crystal truncated cuboctahedron on a substrate, only 6 give diffraction patterns with symmetries different from that of a twinned NP. For higher order index directions, requiring careful tilting, 12 of the 36 〈210〉, 12 of the 24 〈311〉, 12 of the 36 〈410〉 and 24 of the 48 〈321〉directions produce distinguishable patterns. This may be why twinned BCC NPs have never been reported.321 ≤ 4) would indeed not reveal twinning in a BCC NP, potentially leading to incorrect classification. None of the 〈100〉 and 〈111〉 orientations and only 6 of the 12 〈110〉 orientations lead to diagnostic pattern symmetry. Thus, of the 26 probable orientations of a single crystal truncated cuboctahedron on a substrate, only 6 give diffraction patterns with symmetries different from that of a twinned NP. For higher order index directions, requiring careful tilting, 12 of the 36 〈210〉, 12 of the 24 〈311〉, 12 of the 36 〈410〉 and 24 of the 48 〈321〉directions produce distinguishable patterns. This may be why twinned BCC NPs have never been reported.directions produce distinguishable patterns. This may be why twinned BCC NPs have never been reported.

Since there are so few low-index distinguishable directions, care must be taken before concluding that a NP is single crystalline, ideally using one or more of the following strategies. Firstly, twinned NPs can be found by acquiring dark field (DF) TEM images centred on different diffraction spots. In a single crystal, the whole NP will always be seen regardless of the spot used. In a twinned NP, spots are produced by either one or both twins, even in patterns with indistinguishable symmetry, leading to a striking contrast between the twins for some spots. For example, in the [001] direction (Fig. 2d), a DF image centred on the 11[combining macron]0 spot will show both twins, whereas centring on the 110 spot will only show one. Related diffraction contrast may be perceptible in bright field TEM images, most evidently for large NPs in orientations with the beam far from perpendicular to the twin plane. This contrast lacks crystallographic orientation information so should only be used, if seen, as a support in the twinning assessment.

Alternatively, different zone axes could be interrogated by tilting. Those with distinguishable patterns (Fig. 3), can then be found and assigned. This method commonly supplies evidence for twinning in FCC NPs. One could attempt selected area electron diffraction or convergent beam electron diffraction of the larger NPs at a few tilt angles in order to obtain (different if twinned) local diffraction patterns. Another strategy is to record complete three dimensional electron diffraction data^30^ and subsequently extract planes from the reciprocal space that make twinning clear in post-processing.

Direct lattice or atomic symmetry imaging with high-resolution (HR) TEM or STEM can also validly demonstrate twinning in NPs, and has been extensively used for FCC metals. Examples of expected atomic alignment patterns are reported in Fig. 3 and S4.† As with diffraction, we systematically investigated the distinguishable crystallographic directions (Fig. S5†); few show twinning unequivocally. Viewing the NP parallel to the (112) twin produces fringes stacked in an obviously different orientation except for [1[combining macron]1[combining macron]1] and [111[combining macron]], so twinning is best revealed by so tilting the NP and avoiding ); few show twinning unequivocally. Viewing the NP parallel to the (112) twin produces fringes stacked in an obviously different orientation except for [1̄1̄1] and [111̄], so twinning is best revealed by so tilting the NP and avoiding 〈111〉 directions. Images obtained from directions not parallel to the twin plane have varying degrees of differentiation and may be difficult to interpret. Unfortunately, most lattice images shown in support of single crystallinity fall in this non-diagnostic category: Wang 111); few show twinning unequivocally. Viewing the NP parallel to the (112) twin produces fringes stacked in an obviously different orientation except for [1̄1̄1] and [111̄], so twinning is best revealed by so tilting the NP and avoiding 〈111〉 directions. Images obtained from directions not parallel to the twin plane have varying degrees of differentiation and may be difficult to interpret. Unfortunately, most lattice images shown in support of single crystallinity fall in this non-diagnostic category: Wang directions. Images obtained from directions not parallel to the twin plane have varying degrees of differentiation and may be difficult to interpret. Unfortunately, most lattice images shown in support of single crystallinity fall in this non-diagnostic category: Wang *et al.*^15^ and Magnusson *et al.*,^16^ look for fringes along the [001] (simulations in Fig. S4†), while Zhang *et al.*^14^ and Magnusson *et al.*^16^ show (110) (incorrectly identified as (100)) and (111) spacings, respectively, both from unknown directions.

In contrast, FCC NPs are relatively easily identified by their shapes in SEM or TEM, especially in the case of multiply twinned NPs. In addition, knowledge and expectation of twinning along {111} leads to a more stringent analysis and review process, demanding patterns along known diagnostic directions (Fig. S3†) and lattice imaging perpendicular to the known twin plane.

## Conclusions

In conclusion, the shapes and diffraction signatures of twinned BCC NPs can resemble that of their single crystal counterparts, making experimental identification elusive. Yet, twinning is critical in dictating catalytic activity, such that further understanding of its behaviour in BCC NPs is much overdue. We outlined approaches to achieve identification of twinned structures based on diffraction patterns and lattice fringes. These results invite the reconsideration of characterization of new and old BCC NPs. Studies revealing that BCC twins are as common as those of HCP would open a new chapter in the understanding of NP shape; the opposite result would outline a new fundamental differentiation between nanoscale HCP, FCC, and BCC.

### Methods

Analytical, thermodynamic single crystal NP shapes were obtained using the open-source Mathematica-based software Wulffmaker, downloaded from http://pruffle.mit.edu/wulffmaker/. Analytical twinned shapes were obtained by modifying the Wulffmaker code to introduce truncation and reflection on an arbitrary twin plane assumed to form at the centre of the crystal, *i.e.* assuming negligible twin energy compared to surface energies. Numerical, kinetic single crystal and twinned shapes were calculated using Crystal Creator, an open-source Matlab-based software available for download at on.msm.cam.ac.uk/code. Re-entrant enhancements were applied in the kinetic twinned structures to produce more realistic NP shapes. Thickness projections were generated using these kinetic structures. The surface energies used for each shape are reported in Fig. 1 and twinning was modelled on the (112) plane, but applies, of course, for all {112}: in a cubic system, *a, b*, and *c* are equivalent and the choice of specific twin plane is a matter of convention. An equivalent (but rotated) stereogram (Fig. 3a) would be obtained for other {112}-type planes.

Diffraction patterns were computed using SingleCrystal™ (http://www.crystalmaker.com) for a sample of thickness 100 Å at 100 keV. For twinned crystals, two twins were observed along [11[combining macron]0] and [1[combining macron]10] respectively and rotated such that the 112 stereogram poles were aligned, corresponding to (112)-twinning. The twins were rotated simultaneously to view diffraction patterns along chosen directions. Stereograms were generated using the MATLAB toolbox MTEX (; https://mtex-toolbox.github.io/). Atomic alignment patterns were obtained by creating a finite BCC lattice of spheres that was truncated and reflected through a (112) plane in Mathematica.

## Conflicts of interest

There are no conflicts to declare.

## Supplementary Material

Supplementary information
