# Supplementary material for: On the identification of twinning in body-centred cubic nanoparticles
Source: Nanoscale. 2020 Oct 23;12(43):22009–13. doi: 10.1039/d0nr06957d (PMC7689669; doi:10.1039/d0nr06957d)
Supplement: Supplementary file 1 [file NR-012-D0NR06957D-s001.pdf]

Electronic Supplementary Material (ESI) for Nanoscale.
This journal is © The Royal Society of Chemistry 2020

Supplementary information for

On the identification of twinning in body-centred cubic nanoparticles

Elizabeth R. Hopper,[1,2,3] Christina Boukouvala,[1,2] Duncan N. Johnstone,[1] John S. Biggins,[4] Emilie Ringe[1,2*]

1. Department of Materials Science and Metallurgy, University of Cambridge, 27 Charles Babbage Road, Cambridge, United Kingdom, CB3 0FS
2. Department of Earth Sciences, University of Cambridge, Downing Street, Cambridge, United Kingdom, CB2 3EQ
3. Department of Chemical Engineering and Biotechnology, University of Cambridge, Philippa Fawcett Drive, Cambridge, United Kingdom, CB3 0AS
4. Department of Engineering, University of Cambridge, Trumpington Street, Cambridge, United Kingdom, CB2 1PZ

* Corresponding author: er407@cam.ac.uk; +44 (0)1223 334330 (ph.)

Figures and Tables

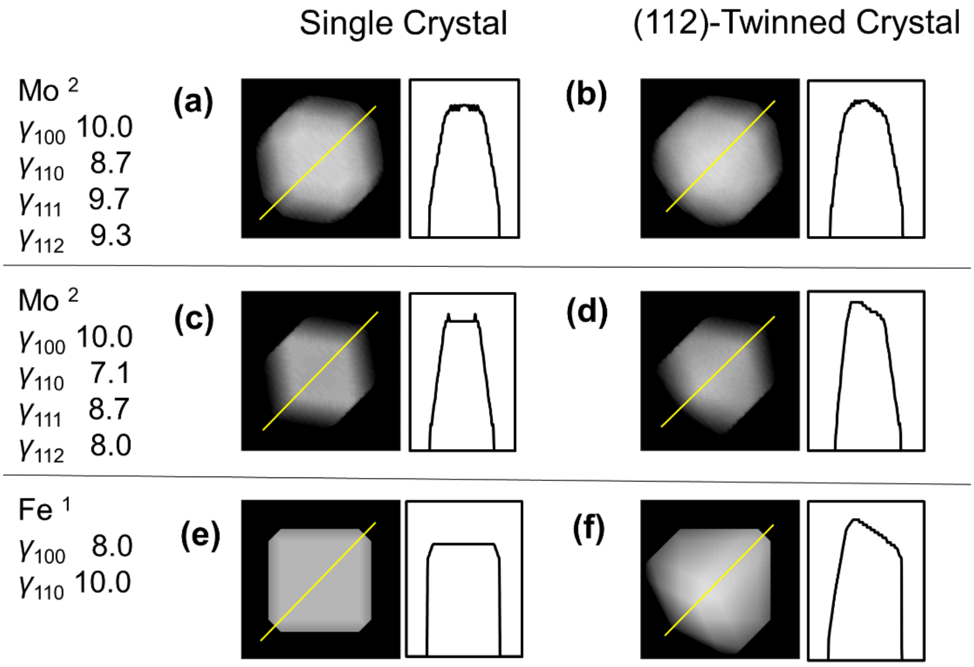

Figure S1: Projected thickness maps as a proxy for HAADF-STEM images and thickness line profile along the yellow line overlay for BCC NPs predicted by the Wulff construction, using the surface energies listed from refs [1] and [2], for (a,c,e) single crystal and (b,d,f) (112)-twinned shapes. (a) and (b) are viewed along the [001] direction and (c,d,e,f) along [110], corresponding to the most likely orientations following deposition. The scattering intensity in the HAADF-STEM varies linearly with thickness, with the thick regions bright and the thin regions dark.

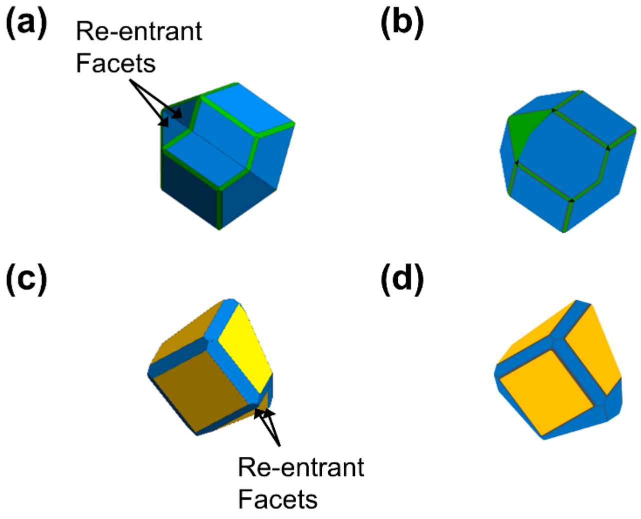

Figure S2: Illustration of re-entrant enhancements for BCC NPs shown in Figure 1. (a) and (c) (112)-twinned shapes using surface energies in refs [2] and [1] with re-entrant facets labelled. (b) and (d) Corresponding shapes with re-entrant kinetic growth enhancement. Facets are coloured by type according to the legend in Figure 1(a).

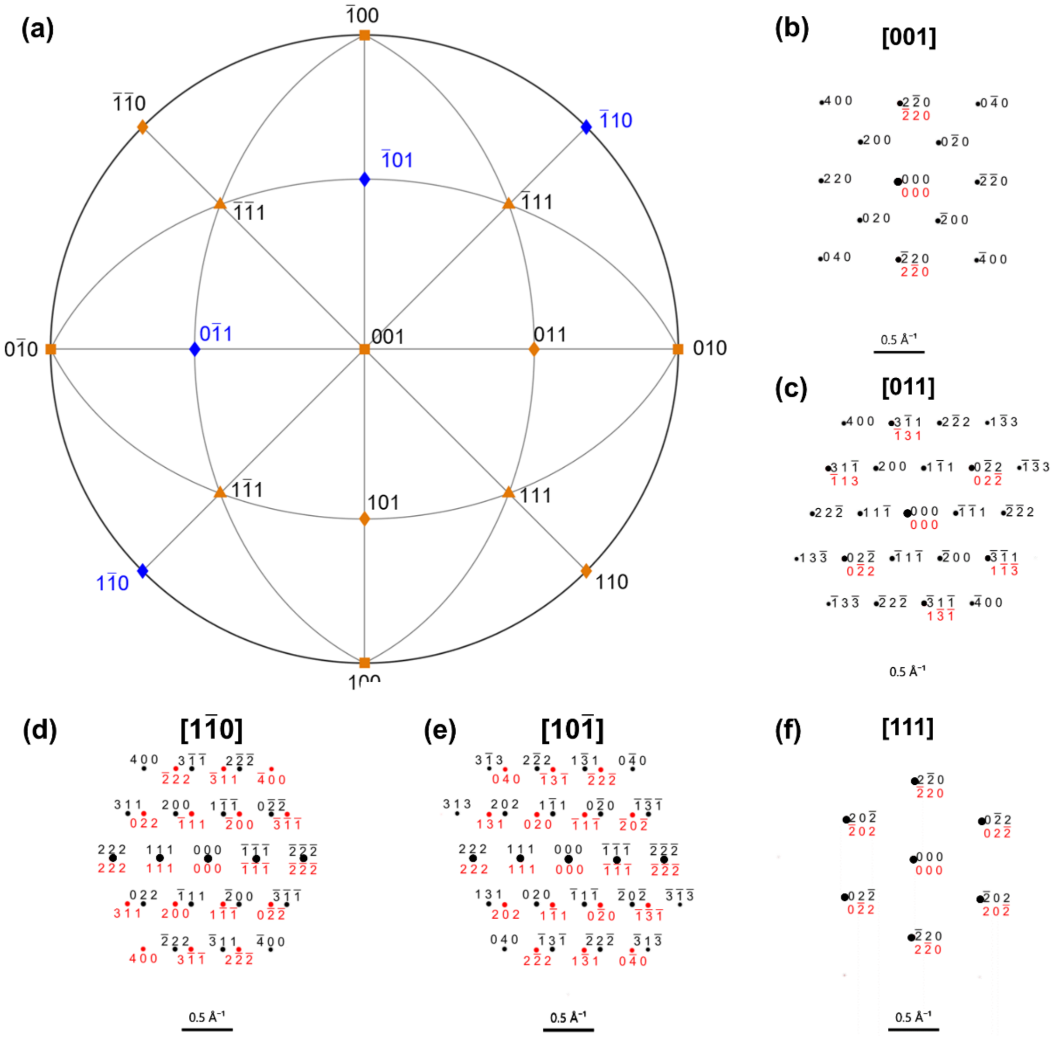

Figure S3. Map and representation of the distinct diffraction patterns enabling the identification of an FCC NP twinned on the (111) plane, along low-index directions and ignoring intensities. (a) Stereographic projection for a cubic particle. Directions giving distinct diffraction spots from the two twins are coloured blue. Directions giving patterns indistinguishable between a twinned and single crystal structure are coloured in orange. (b)-(f) Examples of diffraction patterns along the distinguishable directions indicated in the figure, black and red indicate the patterns of the parent crystal and twin, respectively. The area of the diffraction spots scale with calculated intensity.

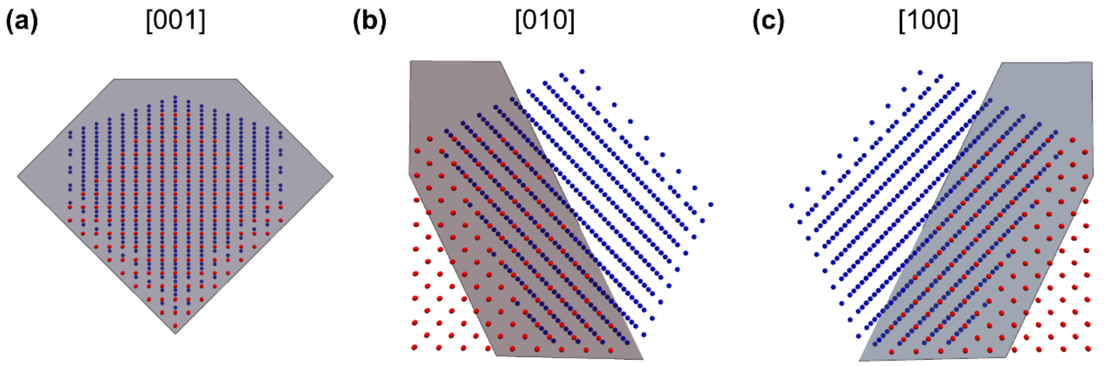

Figure S4. Examples of atomic arrangements (revealed by HR-STEM or TEM) along the [001], [010] and [100] directions for (112)-twinned BCC structures showing continuous fringes across the grain boundary. Blue and red colouring indicate the parent and twinned crystal, respectively.

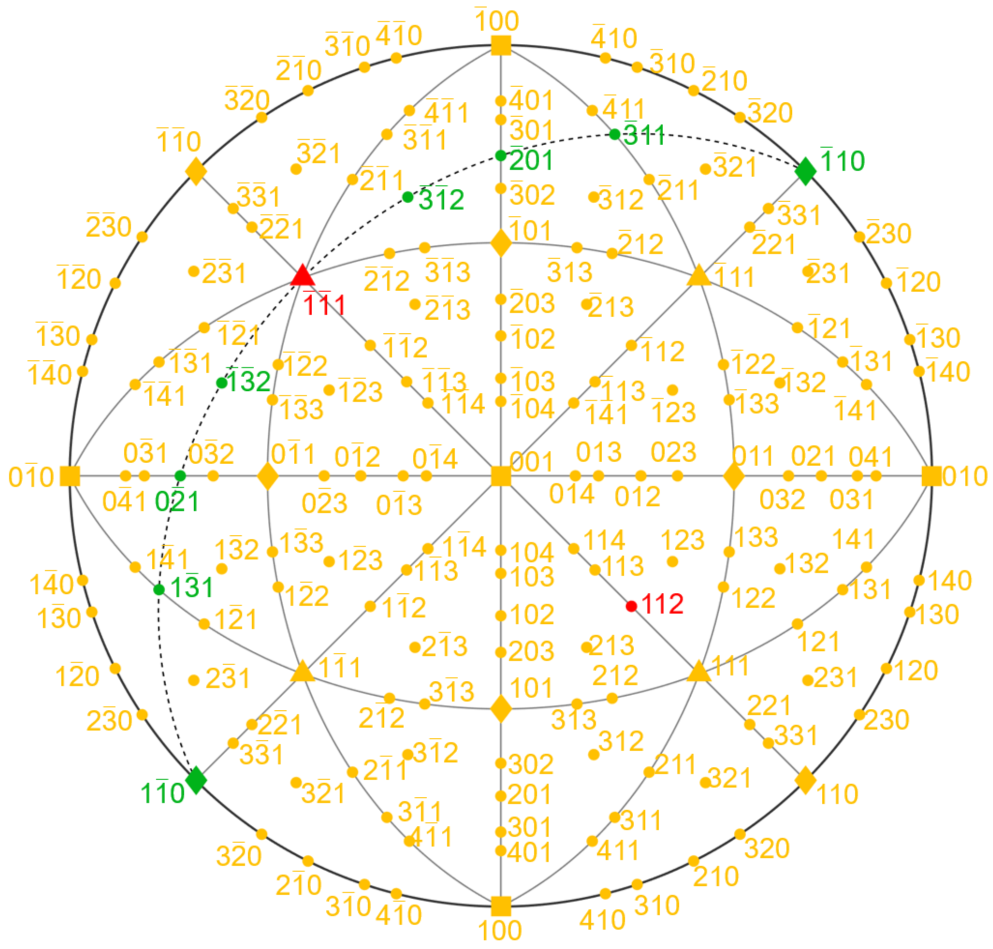

Figure S5. Map of atomic patterns enabling the identification of a BCC NP twinned on the (112) plane, on a stereographic projection for a cubic particle. Directions giving distinct atomic patterns from the two twins are coloured green; directions giving atomic patterns indistinguishable between a twinned and single crystal structure are coloured in red; directions non-parallel to the twin plane, all with varying degrees of differentiation, are coloured orange.

Table S1: Surface areas by facet type for each single crystal and twinned crystal shown in Figure 1.

	{100}		{110}		{111}		{112}	
	Single	Twinned	Single	Twinned	Single	Twinned	Single	Twinned
Fe [3]	1.93	1.84	4.13	4.10	1.34	1.37	0.19	0.20
Mo [2]	0.44	0.44	3.14	3.14	0.04	0.05	2.14	2.14
Mo [2]	0.00	0.00	3.49	3.49	0.00	0.00	0.67	0.67
Fe [1]	4.53	4.53	2.08	2.08	0.00	0.00	0.00	0.00

Table S2: Crystallographic directions with distinguishable diffraction patterns for (112)-twinned BCC NPs.

	**⟨110⟩** (6 out of 12)	**⟨012⟩** (12 out of 36)	**⟨113⟩** (12 out of 24)	**⟨014⟩** (12 out of 36)	**⟨123⟩** (24 out of 48)
Directions	$[1\bar{1}0]$	$[01\bar{2}]$	$[1\bar{1}\bar{3}]$	$[014]$	$[123]$
	$[\bar{1}10]$	$[0\bar{1}2]$	$[\bar{1}13]$	$[0\bar{1}\bar{4}]$	$[\bar{1}\bar{2}\bar{3}]$
	$[011]$	$[10\bar{2}]$	$[1\bar{1}3]$	$[141]$	$[12\bar{3}]$
	$[0\bar{1}\bar{1}]$	$[\bar{1}02]$	$[\bar{1}1\bar{3}]$	$[1\bar{4}\bar{1}]$	$[\bar{1}\bar{2}3]$
	$[101]$	$[0\bar{2}1]$	$[131]$	$[104]$	$[13\bar{2}]$
	$[\bar{1}0\bar{1}]$	$[02\bar{1}]$	$[\bar{1}\bar{3}\bar{1}]$	$[\bar{1}0\bar{4}]$	$[\bar{1}\bar{3}2]$
		$[20\bar{1}]$	$[1\bar{3}1]$	$[1\bar{4}0]$	$[1\bar{3}\bar{2}]$
		$[\bar{2}01]$	$[\bar{1}3\bar{1}]$	$[\bar{1}40]$	$[\bar{1}32]$
		$[120]$	$[3\bar{1}\bar{1}]$	$[401]$	$[213]$
		$[\bar{1}\bar{2}0]$	$[\bar{3}11]$	$[\bar{4}0\bar{1}]$	$[\bar{2}\bar{1}\bar{3}]$
		$[210]$	$[311]$	$[4\bar{1}0]$	$[21\bar{3}]$
		$[\bar{2}\bar{1}0]$	$[\bar{3}\bar{1}\bar{1}]$	$[\bar{4}10]$	$[\bar{2}\bar{1}3]$
					$[2\bar{3}\bar{1}]$
					$[\bar{2}31]$
					$[2\bar{3}\bar{1}]$
					$[\bar{2}31]$
					$[31\bar{2}]$
					$[\bar{3}\bar{1}2]$
					$[3\bar{1}2]$
					$[\bar{3}1\bar{2}]$
					$[32\bar{1}]$
					$[\bar{3}\bar{2}1]$
					$[3\bar{2}1]$
					$[\bar{3}2\bar{1}]$

Simulation Parameters

Table S3: Parameters used for analytical modelling of BCC NP shapes.

	γ_{100}	γ_{110}	γ_{111}	γ_{112}	γ_{twin}
Fe [3]	1.02	1.00	1.04	1.12	0.05
Mo [2]	1.00	0.87	0.97	0.93	0.02
Mo [2]	1.00	0.71	0.87	0.80	0.02
Fe [1]	0.80	1.00			0.02

Table S4: Parameters used for numerical modelling of BCC NP shapes. All calculations were performed with calculation step = 0.25, calculation boundaries = 20, smoothing beta = 2, smoothing box = 3.

	γ_{100}	γ_{110}	γ_{111}	γ_{112}	$\gamma_{re\text{-}entrant}$
Fe [3]	10.2	10.0	10.4	11.2	1.0
Mo [2]	10.0	8.7	9.7	9.3	1.0
Mo [2]	10.0	7.1	8.7	8.0	1.0
Fe [1]	8.0	10.0			1.0

References

1 X. Fan, J. Guan, Z. Li, F. Mou, G. Tong and W. Wang, *J. Mater. Chem.*, 2010, **20**, 1676–1682.

2 J. Che, C. Chan, W. E. Jian and T. Leung, *Phys. Rev. B - Condens. Matter Mater. Phys.*, 1998, **57**, 1875–1880.

3 O. Kovalenko, F. O. Chikli and E. Rabkin, *Scr. Mater.*, 2016, **123**, 109–112.
